# Exosome miR-371b-5p promotes proliferation of lung alveolar progenitor type II cells by using PTEN to orchestrate the PI3K/Akt signaling

**DOI:** 10.1186/s13287-017-0586-2

**Published:** 2017-06-08

**Authors:** Yuan Quan, Zhaohua Wang, Ling Gong, Xinmiao Peng, Melissa A. Richard, Junlan Zhang, Myriam Fornage, Joseph L. Alcorn, Dachun Wang

**Affiliations:** 1 The Brown Foundation Institute of Molecular Medicine for the Prevention of Human Diseases, University of Texas Medical School at Houston, 1825 Pressler Street/IMM 437D, Houston, TX 77030 USA; 2 Tongji Hospital, Tongji Medical College Huazhong University of Science and Technology, Wuhan, Hubei Province 430030 China; 3 Department of Anesthesiology, Ruijin Hospital, Shanghai Jiaotong University School of Medicine, Shanghai, 200025 China; 4 Department of Neuroscience, Baylor College of Medicine, Houston, TX 77030 USA; 5 Department of Internal Medicine, University of Texas McGovern Medical School at Houston, Houston, TX 77030 USA; 6 Department of Pediatrics, University of Texas McGovern Medical School at Houston, Houston, TX 77030 USA

**Keywords:** Exosome miRNAs, MiR-371b-5p, PTEN/PI3K/Akt signaling, Lung alveolar progenitor type II cells, Lung injury

## Abstract

**Background:**

Pathways directing endogenous stem/progenitor cells to restore normal architecture and function of damaged/diseased lungs remain underexplored. Published data have revealed that alveolar progenitor type II cell (ATIIC)-derived signaling promotes re-epithelialization of injured alveoli, yet the underlying mechanism is unknown. Here we aim to define the role of ATIIC-derived exosome miRNA signaling in controlling ATIIC-specific proliferation or differentiation in response to injury.

**Methods:**

Pluripotent stem cell-derived cultures, which contain early lung stem/progenitor populations that can subsequently differentiate into ATIICs, were used as a model for unbiased screening and identification of ATIIC phenotype-specific exosome miRNA signaling, and human induced pluripotent stem cell-derived ATIICs (hiPSC-ATIICs) were employed to examine the molecular basis of key exosome miRNA signaling in promoting ATIIC-specific proliferation. QRT-PCR was performed to examine expression pattern of ATIIC-derived key exosome miRNA in an alveolar injury model and in injured human lungs.

**Results:**

We show that human ATIIC line (A549)-derived exosome miR-371b-5p promotes ATIIC-specific proliferation, but not differentiation, in differentiating cultures of pluripotent stem cells. Using 3′UTR-driven luciferase reporters, we identified PTEN as a direct target of miR-371b-5p. Transfection of miR-371b-5p mimic into hiPSC-ATIICs leads to significantly decreased expression of endogenous PTEN, which stimulates phosphorylation of Akt and its downstream substrates, GSK3β and FOXOs, promoting cell proliferation. While not expressed in normal ATIIC phenotypes, the exosome miR-371b-5p expression is significantly induced after hiPSC-ATIICs or hATIICs (human primary ATIICs) are subjected to bleomycin-induced injury. To rule out that the ATIIC-derived exosome-miRNAs are merely a cell culture phenomenon, we transplanted hiPSC-ATIICs into bleomycin-challenged lungs of mice, and found that the transplanted hiPSC-ATIICs engraft and express exosome miR-371b-5p, along with additional survival of numerous mouse ATIICs in bleomycin-injured lungs. Consistent with these findings, significant levels of exosome miR-371b-5p were also detected in lavage samples of patients with acute pneumonia, but not in those from patients without pulmonary disorders.

**Conclusions:**

Collectively, our data strongly suggest that ATIIC-derived exosome miR-371b-5p may serve as a niche signaling to augment ATIIC survival/proliferation, promoting re-epithelialization of injured alveoli, and thus provide a promising novel target to develop treatment for currently incurable lung diseases.

**Electronic supplementary material:**

The online version of this article (doi:10.1186/s13287-017-0586-2) contains supplementary material, which is available to authorized users.

## Background

Alveolar epithelial type II cells (ATIICs) serve as alveolar progenitor critical for maintaining the integrity of alveolar epithelium, a structure essential for O_2_/CO_2_ exchange. Failure to repair injured alveolar epithelium has been implicated in the development of many life-threatening pulmonary diseases, including acute respiratory distress syndrome, chronic obstructive pulmonary disease, and pulmonary fibrosis. Crosstalk between ATIICs and mesenchymal cells is thought to play an important role in distal lung development, yet niche signaling supporting ATIIC function in response to injury remains largely unknown. We have observed in our previous studies that transplanted ATIICs derived from human pluripotent stem cells not only engraft and differentiate into alveolar epithelial type I cells (ATICs) for repair of injured mouse alveoli, but also provide an appropriate signal microenvironment to promote the repair capacity of endogenous ATIICs [1, 2], suggesting a pivotal role of signal communication between ATIICs or between ATIICs and their upstream stem cells in controlling ATIIC-specific proliferation or differentiation in response to injury.

Exosomes are cell-derived extracellular vesicles containing functional proteins, mRNAs and microRNAs (miRNAs), and serve as a key mediator in intercellular communication [3–5]. Distinct miRNA expression patterns have been found in different tissues, cell types, and developmental stages [6], and shown to play a critical role in controlling various cellular processes such as stem cell self-renewal, proliferation, differentiation, and tissue development [7–16]. Hence, exosomes are likely to be an efficient and robust means to transfer miRNAs and regulate physiological functions of neighboring and distant tissue progenitor cells for tissue regeneration. Because secreting and recycling of vesicle-rich surfactant by ATIICs is a key process in surfactant metabolism in lung [17], we hypothesize that ATIIC-derived exosome miRNAs (Exo-miRs) may be critical to carry cell-to-cell communication necessary for maintaining alveolar homeostasis in response to injury.

MiRNAs, on average 22 nucleotide long, endogenous non-coding RNA molecules, suppress gene expression post-transcriptionally by binding to partially complementary sites within 3′UTR regions of mRNAs in all eukaryotic cells. Over 1000 miRNAs have been identified in human [18], which may target about 60% mammalian genes [19, 20]. Despite mounting evidence demonstrating their importance in numerous cellular processes, little is known about the role of Exo-miR-mediated cellular communications in lung physiology. We here report that a human ATIIC line, A549, expresses exosome miR-371b-5p, which promotes ATIIC-specific proliferation by orchestrating PTEN/PI3K/AKT signaling pathway. While human induced pluripotent stem cell-derived ATIICs (hiPSC-ATIICs) and human primary ATIICs (hATIICs) do not express exosome miR-371b-5p, exosomes isolated from bleomycin (BLM)-treated hiPSC-ATIICs and hATIICs express a significant level of miR-371b-5p. Consistently, lung lavage exosomes of patients with acute pneumonia, but not those without any pulmonary disorders, express miR-371b-5p. In further exploration of the possibility of ATIIC-derived exosome miR-371b-5p serving as a niche signaling, we show the capacity of transplanted hiPSC-ATIICs to engraft and secrete exosome miR-371b-5p in response to injury, along with additional survival of numerous mouse ATIICs in the BLM-injured lungs. These results suggest an essential role of exosome miR-371b-5p-mediated communication between ATIICs in response to lung injury, and that exosome miR-371b-5p may represent a novel therapeutic target for repair of injured alveoli.

## Methods

### Cell cultures

A hiPSC line, hiPSC-26B [2] was cultured on Matrigel-coated plates in differentiation medium (DM) containing 20% FBS (HyClone, Logan, UT, USA), 1% nonessential amino acid, 1 mM L-glutamine, 100 μg/ml penicillin, 100 μg/ml streptomycin, and 20 μg/ml G418 in Knockout DMEM (Gibco, Invitrogen, Waltham, MA, USA) for 14 days, which allows us to select a pure population of functional ATIICs. The hATIICs and mATIICs (mouse primary ATIICs) were prepared as previously described [21, 22]. Human peripheral blood monocytes (hmonos) and NK cells were isolated from peripheral blood samples (Gulf Coast Regional Blood Center, Houston, TX, USA) by using the density gradient centrifugation (Ficoll-Paque) and the NK Cell Isolation Kit (MACS; Miltenyi Biotec, Bergisch Gladbach, Germany), respectively, as previously reported [23].

### Preparation of ATIIC phenotype-derived exosomes and Exo-miRs

A549 cells, hiPSC-ATIICs, and hATIICs were cultured on Matrigel-coated plates with exosome-depleted DM. The medium was collected from each phenotype [A549-, hiPSC-ATIIC-, and hATIIC-conditioned DM or conditioned medium (CM)] in 24 hours, from which exosomes were then isolated using ExoQuick-TC Exosome Precipitation Solution (System Biosciences, Palo Alto, CA, USA) for small RNA (<200 nt) preparation using the mirVana™ miR Isolation kit (Ambion, Austin, TX, USA) by following the manufacturer’s instructions. To prepare exosomes and Exo-miRs from injured ATIICs, we established an ATIC/ATIIC co-culture model. Briefly, 5 × 10^5^ hiPSC-ATIICs were placed in the lower chamber of each six-well transwell plate with DMEM containing 10% FBS and left to spontaneously differentiate for 8 days [1, 2], which allows 100% placed hiPSC-ATIICs to differentiate into ATICs (hiPSC-ATICs) expressing AQP5 and T1α as shown in Additional file 1: Figure S2. Then the derived ATICs were switched to exosome-depleted DM, and 1 × 10^6^ freshly isolated hiPSC-ATIICs or hATIICs were placed in each transwell insert (Matrigel-coated, 0.4 mm pores) with 1 ml exosome-depleted DM. Next day, various doses (10, 20, 30, and 40 μg/ml) of BLM were added to the medium to induce injury for 24 hours before exosome and Exo-miR isolation.

### Exosome miRNA profiling analysis

Exo-miRs were prepared for array analysis (performed by LC Sciences, Houston, TX, USA). The human miRNA array (MRA-1001) that targets all known human mature miRNAs in the miRNA database (Release 18, hrrp://microrna.sanger.ac.uk) was used. The miRNA expression levels identified in the analyses were validated by QRT-PCR for consistency.

### Construction of miRNA-inhibitor vectors

For each miRNA-inhibitor vector, a double-stranded oligonucleotide was designed to contain a targeting motif for specific binding to an individually selected miRNA molecule, with *Xho* I or *Xba* I overhang at each end, and was then cloned into Sal I and Xba I sites downstream of the U6 promoter in the pSuppressorNeo vector as shown in Fig. 2c. The sequences of targeting motifs are listed in the figure legends.

### Examination of the effect of ATIIC-derived signaling on ATIIC-specific differentiation or proliferation

To examine the effect of ATIIC phenotype-derived signaling on ATIIC-specific differentiation or proliferation in the cultures of pluripotent stem cells, a human embryonic stem cell (hESC) line, SPCP/NEO74 [24], which harbor ATIIC-specific surfactant protein C (SPC) promoter/neomycin^R^ (SPCP/NEO^R^) transgene, was cultured on Matrigel-coated six-well plates in DM for 6 days, and then some of the differentiating cultures were switched to A549-CM, hiPSC-ATIIC-CM, hATIIC-CM, or DM containing ATIIC phenotype-derived exosomes for 6 or 10 days, with the medium changed every day. Exosomes isolated from 5 × 10^6^ each ATIIC phenotype were added into one corresponding well for the study. In order to test the effect of A549-derived Exo-miRs on ATIIC-specific proliferation, the hESC-derived cultures were co-transfected with A549-derived Exo-miRs (1.0 μg) and one selected individual miRNA inhibitor vector (0.5 μg) on days 6 and 12 using Lipofectamine® RNAiMAX Transfection Reagent (Invitrogen). To determine the content of the derived SPC-expressing cells in the differentiated cultures of hESCs, the differentiated cells were stained with 1:500 diluted anti-human proSPC antibody (Chemicon, Temecula, CA, USA) on days 12 and 16. The number of SPC-positive cells was counted per 1000 cells based on 4′,6-diamidino-2-phenylindole (DAPI, Biostatus, Loughborough, UK) staining on each plate. To examine the capacity of miR-371b-5p to induce ATIIC proliferation, the G418-selected hiPSC-ATIICs in six-well plates were transfected with various doses (50, 100, and 150 pmols/well) of miR-371b-5p mimic (Ambion) and then incubated with 10 μM bromodeoxyuridine (BrdU) (Abcam, Cambridge, MA, USA) for 12 hours to label the proliferating cells. Proliferation was assessed 2 days after transfection using the CellTiter 96®AQ_ueous_ One Solution Cell Proliferation Assay Kit (Promega, Madison, WI, USA).

### Immunofluorescent staining and Western blot analysis

Differentiated hESCs and hiPSC-ATIICs were washed twice with PBS and fixed in 4% paraformaldehyde for 15 minutes. Washed cells were permeabilized in 0.5% Triton X (Sigma-Aldrich, St. Louis, MO, USA) for 30 minutes before being blocked in 5% goat serum for 2 hours. Cells were then stained with 1:500 diluted rabbit anti-human proSPC antibody and/or mouse anti-BrdU antibody (1:100, Abcam), and visualized with Alexa Fluor 546- or 488-conjugated goat anti-rabbit IgG or Alexa Fluor 546-conjugated goat anti-mouse IgG (1:1000, Molecular Probes, Eugene, OR, USA) with 1:1000 diluted DAPI counterstaining. In addition, the rabbit anti-human proSPC and mouse anti-human nuclei monoclonal antibody (Chemicon) were used for immunofluorescent staining of tissue sections of BLM-challenged lungs transplanted with hiPSC-ATIICs as previously reported [1, 2]. For Western blot analysis, protein extracts (20 μg) from miR-371b-5p mimic-treated hiPSC-ATIICs were prepared. Rabbit anti-human Akt/pAkt, Erk/pErk, GSK3β/pGSK3β, FOXO3/pFOXO3, FOXO1/pFOXO1, PTEN, and GAPDH (Cell Signaling, Danvers, MA, USA) were used in the study. In addition, 10 μg ATIIC phenotype-derived exosomal proteins were analyzed for exosome marker expression by using rabbit anti-TSG101, mouse anti-CD63 and mouse anti-HSP70 antibody (Abcam).

### Dual-luciferase miRNA target reporter assay

To generate the dual-luciferase miRNA target reporter vector, a double-stranded oligonucleotide containing wild-type (wt) PTEN 3′UTR sequence with a predicted miR-371b-5p target site or mutant PTEN 3′UTR sequence was cloned into Pmel and XbaI sites in pmirGLO Dual-Luciferase miRNA Target Expression Vector (Promega, Fig. 4b). The hiPSC-ATIICs of 70% confluence in 24-well plates were co-transfected with the dual-luciferase miRNA target reporter vector (0.3 μg) and miR-371b-5p mimic (15 pmol/well). Cell extracts were prepared 24 hours after transfection, and the luciferase activity was measured using the Dual-Luciferase Reporter Assay System (Promega).

### QRT-PCR

0.5 μg total cellular/exosome RNA (0.1 μg for human/mouse lavage exosome RNA) were prepared for QRT-PCR analysis using TaqMan One-Step RT-PCR Master Mix Kit (AB Applied Biosystems, Foster City, CA, USA) as previously described [1], using the following primers and probes: (1) miR-371b-5p primers (ABM Inc., Richmond, BC, Canada); (2) SPC forward primer (5′-GTC CTC ATC GTC GTG GTG ATT-3′), SPC reverse primer (5′-CGT GTG TTT CTG GCT CAT GTG -3′), and SPC probe (5′- 6-FAM-AGA CCC ATG AGC AGG GCT CCC -TAMRA-3′); (3) PTEN forward primer (5′- ACC CAC ACG ACG GGA AGA CA -3′), PTEN reverse primer (5′- CTG TTT GTG GAA GAA CTC TAC TTT GAT ATC AC -3′), and PTEN probe (5′-6-FAM-CAT GTA CTT TGA GTT CCC TCA GCC GTT ACC TGT G-TAMRA-3′); and (4) 18S forward primer (5′-TAA CGA ACG AGA CTCTGG CAT-3′), 18S reverse primer (5′-CGG ACA TCT AAG GGC ATC ACA G-3′), and 18S probe (5′-FAM-TGG CTG AAC GCC ACT TGT CCC TCT AA-TAMRA-3′).

### Statistical analysis

Data were analyzed using one-way ANOVA test and the results with pairwise *P* values less than 0.05 were considered statistically significant.

## Results

### A549 cell-derived exosome miR-371b-5p promotes ATIIC-specific proliferation

**Fig. 1:**
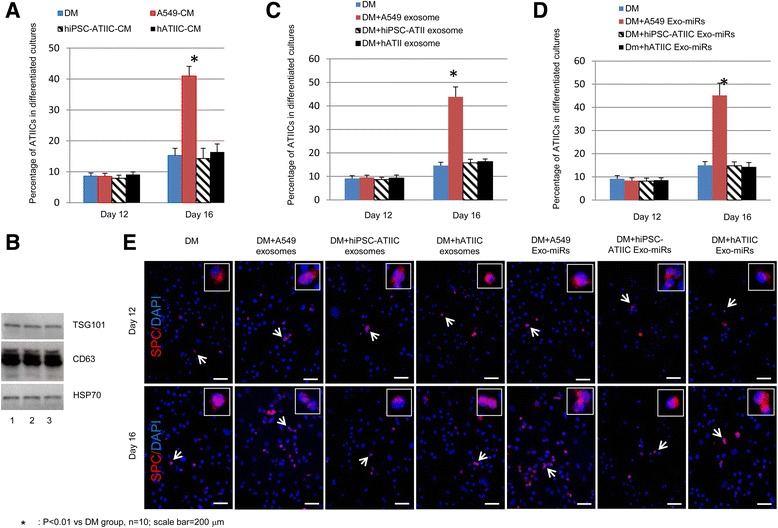
A549-derived Exo-miRs promote ATIIC-specific proliferation. Bar graph representation of the content of SPC-expressing cells in the differentiated cultures of hESCs to show the effects of each ATIIC phenotype-derived CM (**a**), exosomes (**c**) or Exo-miRs (**d**) on ATIIC-specific differentiation (day 12) and proliferation (day 16). **b** Western blotting of TSG101, CD63 and HSP70 in exosomes derived from cultured A549 (1), hiPSC-ATIICs (2) and hATIICs (3). **e** Representative immunofluorescent staining of the SPC-expressing cells (*red*) in the differentiated cultures of hESCs after treatment with A549-, hiPSC-ATIIC-, or hATIIC-derived exosomes or Exo-miRs on days 12 and 16. *ATIICs* alveolar epithelial type II cells, *CM* conditioned medium, *DAPI* 4′,6-diamidino-2-phenylindole, *DM* differentiation medium, *Exo-miRs* exosome miRNAs, *hATIICs* human primary ATIICs, *hiPSC-ATIICs* human induced pluripotent stem cell-derived ATIICs

**Fig. 2:**
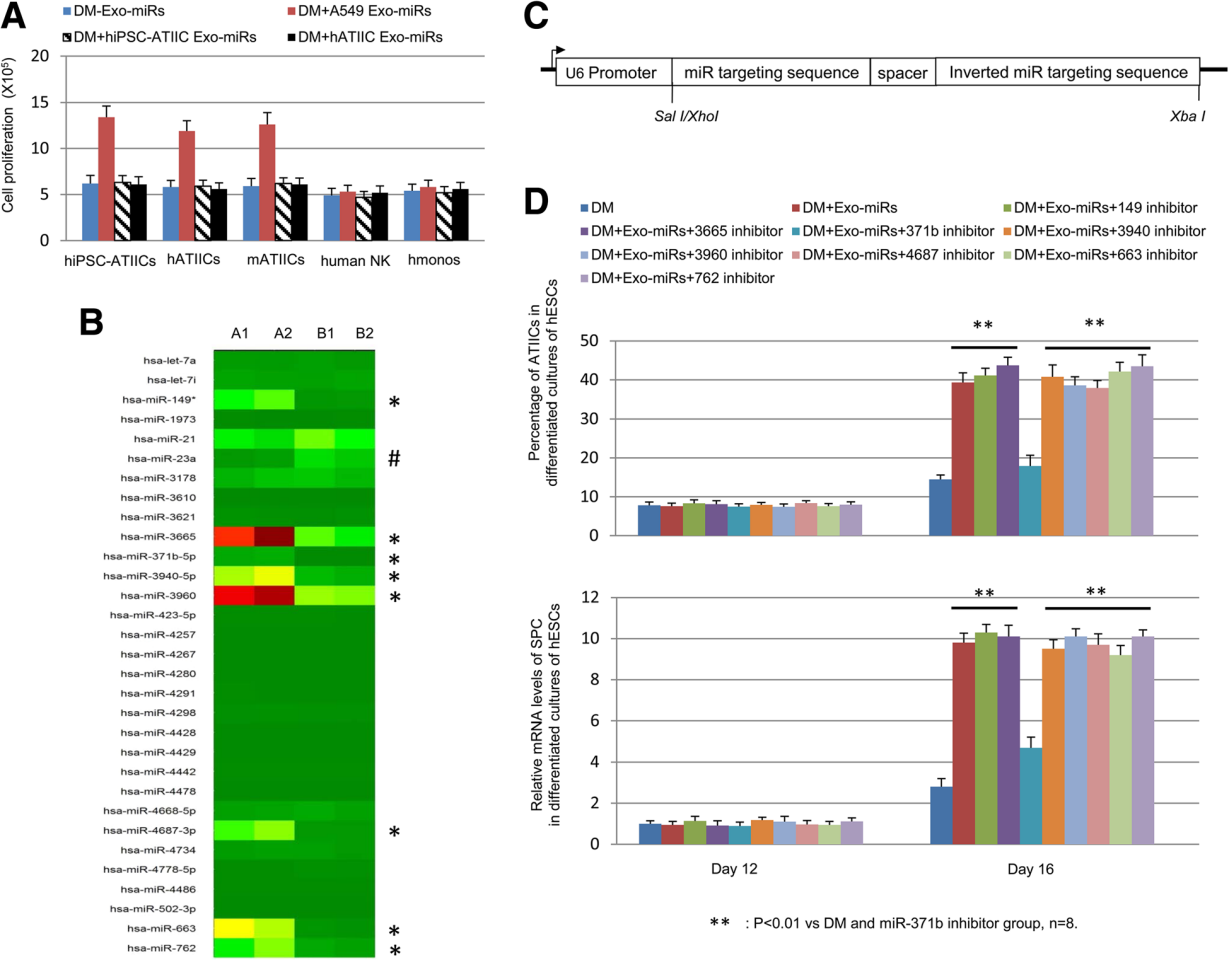
A549-derived exosome miR-371b-5p promotes ATIIC-specific proliferation. **a** Histogram representation of the number of viable cells in the cultures of hiPSC-ATIICs, hATIICs, mATIICs, human NK cells, and human monocytes after being treated with ATIIC-phenotype-specific Exo-miRs. **b** ATIIC-phenotype-specific Exo-miR expression patterns were represented by color heat maps (A: A549 cells, B: hiPSC-ATIICs). Nine Exo-miRs showed significantly differential expression between A549 cells and hiPSC-ATIICs (marked with * or #), eight of which (marked with *) showed significantly elevated expression in A549 cells. **c** Schematic structure of miRNA-inhibitor vectors. Each vector harbors a miRNA targeting motif corresponding to one of the eight selected miRNA sequences. The targeting motif in the vector is separated from its inverted repeat sequence by a spacer of 8 nt. The diagram is drawn to show relevant information only, not scaled proportionally according to the sequence length. The sequences of targeting motifs used to build the miRNA-inhibitor vectors are listed below: (1) aaagtgccgccatcttttgagt for miR-371b-5p, (2) gcacagcccccgtccctccct for miR-149, (3) cgccgccccgcacctgct for miR-3665, (4) cagagcccgccccaacccac for miR-3940-5p, (5) cccccgcctccgccgccgcc for miR-3960, (6) gcctgccccctccaacagcca for miR-4687-3p, (7) gcggtcccgcggcgccccgcct for miR-663, and (8) gctcggccccggccccagcccc for miR-762. **d** The content of SPC-expressing cells (*top panel*) and the relative SPC expression levels (QRT-PCR, *bottom panel*) in the differentiated cultures of pluripotent stem cells were analyzed to show the effects of A549-derived Exo-miRs on ATIIC-specific differentiation (day 12) and proliferation (day 16) after one of the A549-specific Exo-miRs had been inhibited. *ATIICs* alveolar epithelial type II cells, *DM* differentiation medium, *Exo-miRs* exosome miRNAs, *hATIICs* human primary ATIICs, *hESCs* human embryonic stem cells, *hiPSC-ATIICs* human induced pluripotent stem cell-derived ATIICs, *hmonos* human peripheral blood monocytes, *mATIICs* mouse primary ATIICs, *SPC* surfactant protein C

Based on literature and our previous studies [1, 2], we hypothesize that ATIIC-derived Exo-miRs may be crucial in mediating cell-to-cell communication that supports ATIIC-specific proliferation and/or differentiation in response to lung injury. To test this, we first used pluripotent stem cell-derived cultures as models to examine ATIIC-signaling derived from A549, hiPSC-ATIICs, and hATIICs. As previously reported [2], an hiPSC line, hiPSC-26B, was used to prepare a pure population of hiPSC-ATIICs (>99%, Additional file 1: Table S1 and Figure S1), which can be induced to differentiate into ATICs (Additional file 1: Figure S2). As described in “Methods”, a hESC line, SPCP/NEO.74 [24], was subjected to spontaneous differentiation in DM for 6 days, and then the hESC-derived cultures, which conceivably contain many tissue cell lineages including early lung stem/progenitor populations, were switched to A549-CM, hiPSC-ATIIC-CM, or hATIIC-CM for 6 or 10 days before immunostaining for ATIIC-specific SPC expression. In comparison with hESC-derived cultures in DM on day 12, in which approximately 9% cells expressed SPC protein, the hESC-derived cultures treated with A549-CM, hiPSC-ATIIC-CM, or hATIIC-CM for 6 days did not show an increased content of SPC-expressing cells (Fig. 1a). Since the hESCs contain the SPCP/NEO^R^ transgene, these SPC-expressing cells should express NEO^R^. Thus, we were able to isolate and characterize these SPC-expressing cells from the hESC-derived cultures using G418 selection strategy [24]. As we previously reported [2, 24], all these SPC-expressing cells also expressed other surfactant proteins and lamella bodies, but not CCSP on day 12 (Additional file 1: Figure S3), representing a pluripotent stem cell-derived mature ATIIC phenotype. Together, these results suggest that A549 cells, hiPSC-ATIICs, and hATIICs do not derive an active signaling to promote ATIIC-specific differentiation from early lung stem/progenitor cells. However, 10-day treatment with A549-CM, but not with hiPSC-ATIIC-CM or hATIIC-CM, resulted in an enriched SPC-expressing cell population (41%), yet only approximately 15% of cells in hESC-derived cultures in DM expressed SPC on day 16 (Fig. 1a). This enrichment of SPC-expressing cells in the spontaneously differentiated hESC cultures indicated that the early-derived ATIICs in cultures had subsequently been induced to proliferate by A549-derived factors. To further test if Exo-miRs are the key factors to convey A549 cell-derived signaling, exosomes as well as Exo-miRs were isolated from A549-CM, hiPSC-ATIIC-CM, and hATIIC-CM, respectively, and used to treat hESC-derived cells. The isolated exosomes, verified by expression of exosome-specific markers TSG101, CD63, and HSP70 (Fig. 1b) [25–27], were added into hESC-derived cultures in exosome-depleted DM on day 6. As expected, we found that after 10-day treatment with A549-derived exosomes, but not with hiPSC-ATIIC- or hATIIC-derived exosomes, the hESC-derived SPC-expressing cells were enriched to the degree similar to that observed in hESC-derived cultures treated with A549-CM, suggesting an exosome-mediated communication between ATIICs (Fig. 1c and e). Interestingly, SPC-expressing cells were similarly enriched in hESC-derived cultures on day 16 after the differentiating hESCs were transfected with A549-derived Exo-miRs, but not with hiPSC-ATIIC- or hATIIC-derived Exo-miRs, on days 6 and 12 (Fig. 1d and e). Such A549 Exo-miR-mediated cell proliferation was observed in the cultures of purified hiPSC-ATIICs, hATIICs, and mATIICs, but not in the cultures of human NK cells or hmonos (Fig. 2a). Collectively, these results demonstrate that an ATIIC-derived signaling directs ATIIC-specific proliferation, but not ATIIC-specific differentiation from pluripotent stem cells, via Exo-miR-mediated cell-to-cell communication in vitro, while the normal ATIIC-phenotypes, hiPSC-ATIICs, and hATIICs, are not active in this process.

To further identify the A549-derived key Exo-miR regulating ATIIC-specific proliferation, microarray analysis was performed to characterize A549-specific Exo-miR signature. Purified hiPSC-ATIICs, which exhibit hATIIC-specific miRNA expression pattern (Additional file 1: Figure S1), were used as control. The identified miRNA expression levels were validated by QRT-PCR for consistency. We found 31 Exo-miR molecules secreted by A549 cells. Among these, nine Exo-miRs were differentially expressed between A549 cells and hiPSC-ATIICs (Fig. 2b, indicated with * or #), eight of which (marked with *) expressed at significantly higher levels in A549 cells than in hiPSC-ATIICs. To screen these A549-derived Exo-miRs in an unbiased manner, we generated eight miRNA-inhibitor vectors (Fig. 2c) by using the pSuppressorNeo vector backbone [28], each designed to express a targeting motif for specific binding to one of the eight highly expressed A549 Exo-miR molecules. We delivered A549-derived Exo-miRs together with one miRNA-inhibitor vector into hESC-derived cells on days 6 and 12, and found that in all cases, A549-derived Exo-miRs lost their capacity to promote ATIIC-specific proliferation when the vector expressing miR-371b-5p inhibitor was co-transfected, as evidenced by a significantly reduced content of SPC-expressing cells, along with a greatly decreased SPC expression level in the cultures (Fig. 2d). Although it could be a concern that the isolated Exo-miR samples may be contaminated with a small amount of mRNAs, our result clearly rules out the role of exosome mRNAs, and suggests a pivotal role of exosome miR-371b-5p-mediated communication between ATIICs in supporting ATIIC-specific proliferation.

**Fig. 3:**
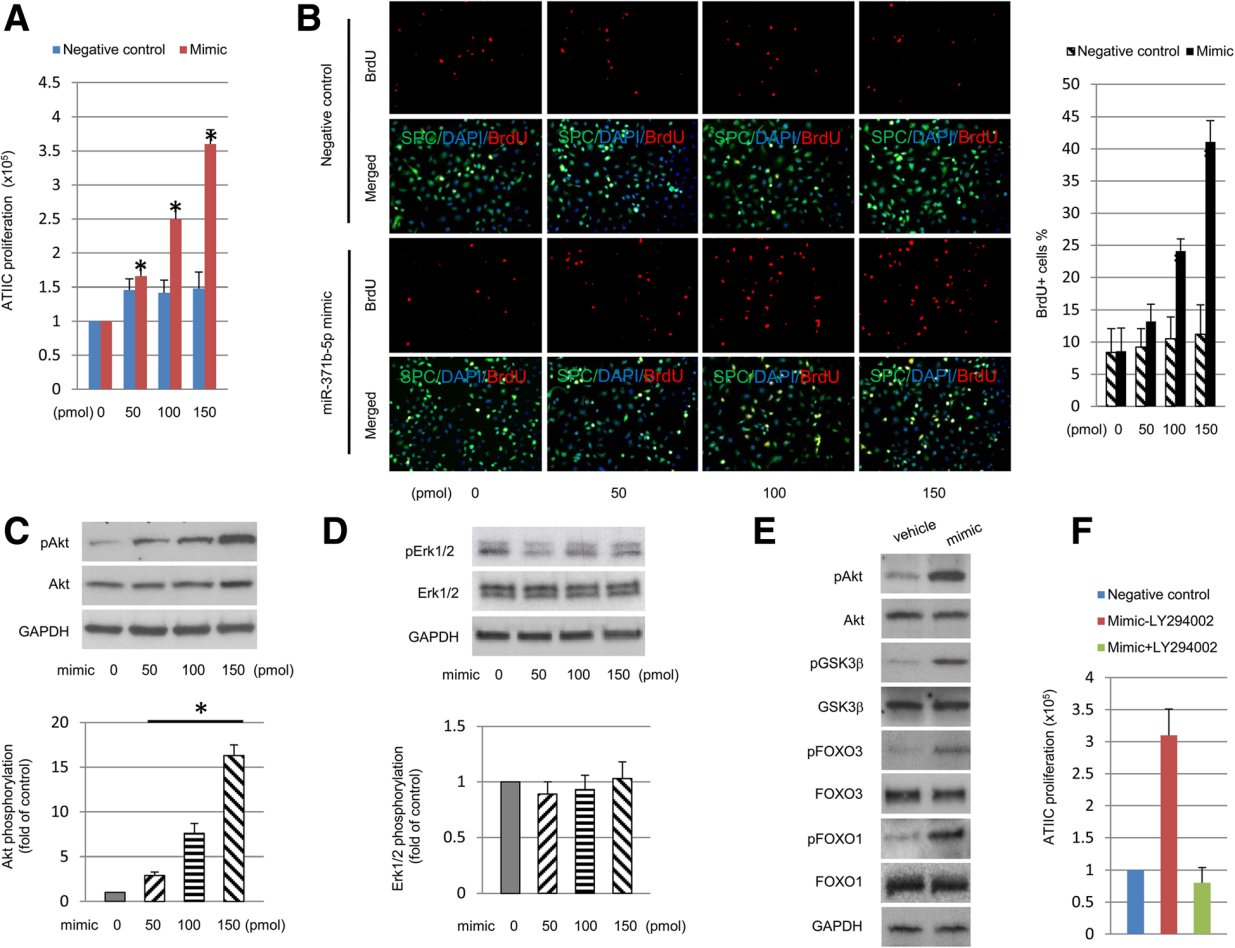
MiR-371b-5p activates PI3K/Akt pathway in hiPSC-ATIICs. The cultured hiPSC-ATIICs were transfected with various doses of miR-371b-5p mimic as indicated. **a** Histogram representation of the number of viable cells in the cultures of miR-371b-5p mimic-treated hiPSC-ATIICs. **b** The miR-371b-5p mimic-transfected hiPSC-ATIICs were treated with BrdU for 12 hours to label the proliferating cells and then were immunostained with mouse anti-BrdU (*red*) and rabbit anti-SPC antibody (*green*). The BrdU-stained images were merged with SPC-stained images (*left panel*), and the BrdU^+^cells were counted and presented as the percentage of total cells in the miR-371b-5p mimic-treated cultures (*right panel*). **c** and **d** Western blot analysis of expression of Akt/pAkt and Erk/pErk in miR-371b-5p mimic-transfected hiPSC-ATIICs (*top panel*). The pAkt and pErk expression levels were quantified and presented as folds of control after being normalized to Akt and Erk, respectively (*bottom panel*). **e** Western blotting of Akt/pAkt, GSK3β/pGSK3β, FOXO3/pFOXO3, and FOXO1/pFOXO1 of hiPSC-ATIICs treated with miR-371b-5p mimic (150 pmol) using GAPDH as internal control. **f** The miR-371b-5p mimic-transfected hiPSC-ATIICs were incubated for 24 hours with and without LY294002 (25 μM). The number of viable cells in the cultures was counted 2 days after transfection. *ATIICs* alveolar epithelial type II cells, *BrdU* bromodeoxyuridine, *DAPI* 4′,6-diamidino-2-phenylindole, *SPC* surfactant protein C

To further confirm the critical role of miR-371b-5p in promoting ATIIC-specific proliferation, purified hiPSC-ATIICs (Additional file 1: Table S1 and Figure S1), which we have previously demonstrated as a functional ATIIC phenotype both in vitro and in vivo [2], were transfected with increasing concentrations (50, 100, and 150 pmols) of miR-371b-5p mimic or negative control in six-well plates and then treated with 10 μM BrdU overnight to label the proliferating cells as described in “Methods”. Many colonies were observed (data not shown), and the number of viable hiPSC-ATIICs significantly increased, along with increased percentages of BrdU-labeled SPC-expressing hiPSC-ATIICs in a dose-dependent manner 2 days after treatment with miR-371b-5p mimic (Fig. 3a and b). This showed that transfection of miR-371b-5p mimic resulted in a dose-dependent hiPSC-ATIIC proliferation with a minimal cytotoxicity.

### Activation of PI3K/Akt signaling pathway in miR-371b-5p mimic-treated hiPSC-ATIICs

To understand the molecular basis of miR-371b-5p-mediated ATIIC-specific proliferation, we examined if PI3K/Akt and/or MAPK/Erk, the two major proliferation-linked signaling pathways [29], are activated in miR-371b-5p mimic-treated hiPSC-ATIICs. We first tested Akt phosphorylation as an index of PI3K/Akt pathway activation. HiPSC-ATIICs were transfected with various doses of miR-371b-5p mimic, and after 24 hours, phosphorylation was examined using anti-phosphorylated Akt kinase antibody. The miR-371b-5p mimic-treated hiPSC-ATIICs expressed significantly increased levels of phosphorylated Akt kinase protein in a dose-dependent manner (Fig. 3c), suggesting the presence of PI3K-dependent Akt kinase activation [30, 31]. To assess the MAPK/Erk signaling pathway, we analyzed the phosphorylation status of Erk1/2 in the miR-371b-5p mimic-transfected hiPSC-ATIICs and found that these cells did not show a significantly increased expression level of phosphorylated Erk1/2 (Fig. 3d). Next we examined phosphorylation of Akt kinase substrates, and found that treatment with 150 pmol miR-371b-5p mimic stimulated Akt kinase activation, which in turn phosphorylated endogenous GSK3β, FOXO3A, and FOXO1A, as evidenced by significantly increased phosphorylation levels of these proteins (Fig. 3e). These data demonstrate that the PI3K/Akt signaling pathway, but not MAPK/Erk signaling pathway, is activated in miR-371b-5p mimic-treated hiPSC-ATIICs. To further confirm this finding, LY294002 (25 μM), a PI3K inhibitor, was added into the cultures of miR-371b-5p mimic-treated hiPSC-ATIICs for 24 hours. This treatment inhibited phosphorylation of Akt kinase and its substrates (data not shown) and completely blocked the miR-371b-5p mimic-induced cell proliferation (Fig. 3f). Taken together, our data demonstrate that treatment with miR-371b-5p mimic results in activation of PI3K/Akt signaling pathway in hiPSC-ATIICs. As the FOXO factors serve as mediators of apoptosis and cell-cycle arrest [32], activated Akt kinase that phosphorylates and inhibits transcriptional activity of FOXO factors may account for miR-371b-5p-induced cell proliferation.

### MiR-371b-5p mimic promotes ATIIC proliferation by targeting PTEN

**Fig. 4:**
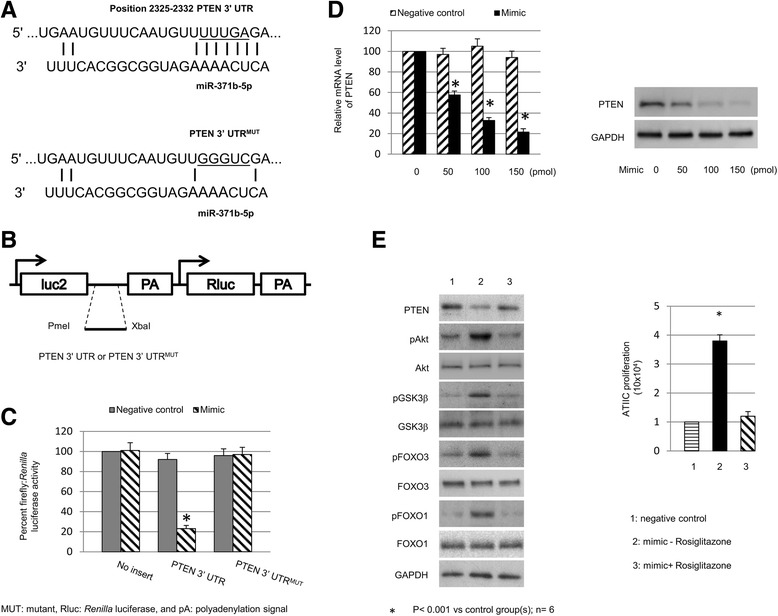
MiR-371b-5p utilizes PTEN to orchestrate the PI3K/Akt signaling in hiPSC-ATIICs. **a** The predicted RNA duplexes of miR-371b-5p target site in the 3′UTR of PTEN (*top panel*). The underlined nucleotides (UUUGA) in the target site (*top panel*) were replaced with GGGUC in the mutant 3′UTR construct (*bottom panel*). **b** Schematic structure of dual-luciferase miRNA target reporter, in which wt or mutant PTEN 3′UTR was inserted into the Pmel and XbaI sites downstream of firefly luciferase (Luc2). **c** Normalized firefly luciferase activity using the dual-luciferase miRNA target reporter with a wt or mutant PTEN 3′UTR in hiPSC-ATIICs co-transfected with miR-371b-5p mimic. **d** QRT-PCR analysis of mRNA expression of PTEN in hiPSC-ATIICs transfected with various doses of miR-371b-5p mimic as indicated. The expression levels were normalized to 18 s and presented as percentages of control (*left panel*). PTEN protein expression of each group of miR-371b-5p mimic-transfected hiPSC-ATIICs was analyzed by Western blot using GAPDH as an internal control (*right panel*). **e** The miR-371b-5p mimic-transfected hiPSC-ATIICs were incubated for 24 hours with and without Rosiglitazone (20 μM). The protein extracts were prepared for Western blot analysis of PTEN, Akt/pAkt, GSK3β/pGSK3β, FOXO3/pFOXO3, and FOXO1/pFOXO1 with GAPDH as control (*left panel*), and the number of viable cells in the cultures was counted 2 days after transfection (*right panel*). *ATIICs* alveolar epithelial type II cells

PTEN serves as a key negative regulator of PI3K/Akt signaling pathway. Its lipid phosphatase activity converts the phosphatidylinositol-(3,4,5)-trisphosphate (PIP3) into phosphatidylinositol-(4,5)-bisphosphate [33, 34], subsequently decreasing PIP3 level and thereby inhibiting phosphorylation and activation of Akt kinase [35]. As PTEN is one of the miR-371b-5p targets predicted by TargetScan [36] and miRDB [37], we aimed to examine whether miR-371b-5p works through PTEN to orchestrate PI3K/AKT signaling pathway for promoting ATIIC-specific proliferation. We used the dual-luciferase miRNA target reporter vectors, which express the primary firefly luciferase reporter fused with wt or mutant PTEN 3′UTR and the control *Renilla* luciferase reporter (Fig. 4a and b), to test whether PTEN is a target of miR-371b-5p. In comparison to controls, the activity of the firefly luciferase reporter fused with wt PTEN 3′UTR, but not the activity of those fused with mutant PTEN 3′UTR, was significantly suppressed in the hiPSC-ATIICs when miR-371b-5p mimic was co-transfected (Fig. 4c). This indicates that the firefly luciferase reporter mRNA becomes a target of miR-371b-5p when fused with wt PTEN 3′UTR. In addition, we observed that the expression levels of endogenous PTEN mRNA and protein significantly decreased in a dose-dependent manner when hiPSC-ATIICs were transfected with miR-371b-5p mimic (Fig. 4d). These data demonstrate that miR-371b-5p regulates PTEN expression post-transcriptionally by directly targeting its 3′UTR. We further used the PPARγ agonist Rosiglitazone, which can activate PTEN expression transcriptionally [38, 39], to treat hiPSC-ATIICs for 24 hours after transfection with miR-371b-5p mimic. As mRNA of PTEN can be significantly induced by Rosiglitazone in a dose-dependent manner (data not shown), Rosiglitazone is able to compete the miR-371b-5p mimic-mediated degradation of PTEN mRNA in hiPSC-ATIICs. As expected, the miR-371b-5p mimic-treated hiPSC-ATIICs in the cultures with Rosiglitazone (20 μM) expressed PTEN at a level comparable to that expressed by control hiPSC-ATIICs (Fig. 4e, left). Meanwhile, the treatment with Rosiglitazone inhibited phosphorylation of Akt kinase and its downstream targets in the miR-371b-5p mimic-treated hiPSC-ATIICs (Fig. 4e, left), and blocked the miR-371b-5p mimic-induced cell proliferation (Fig. 4e, right). Taken together, our results demonstrate that miR-371b-5p promotes ATIIC-specific proliferation by orchestrating the PTEN/PI3K/AKT signaling pathway.

### ATIICs express exosome miR-371b-5p in vitro and in vivo in response to injury

**Fig. 5:**
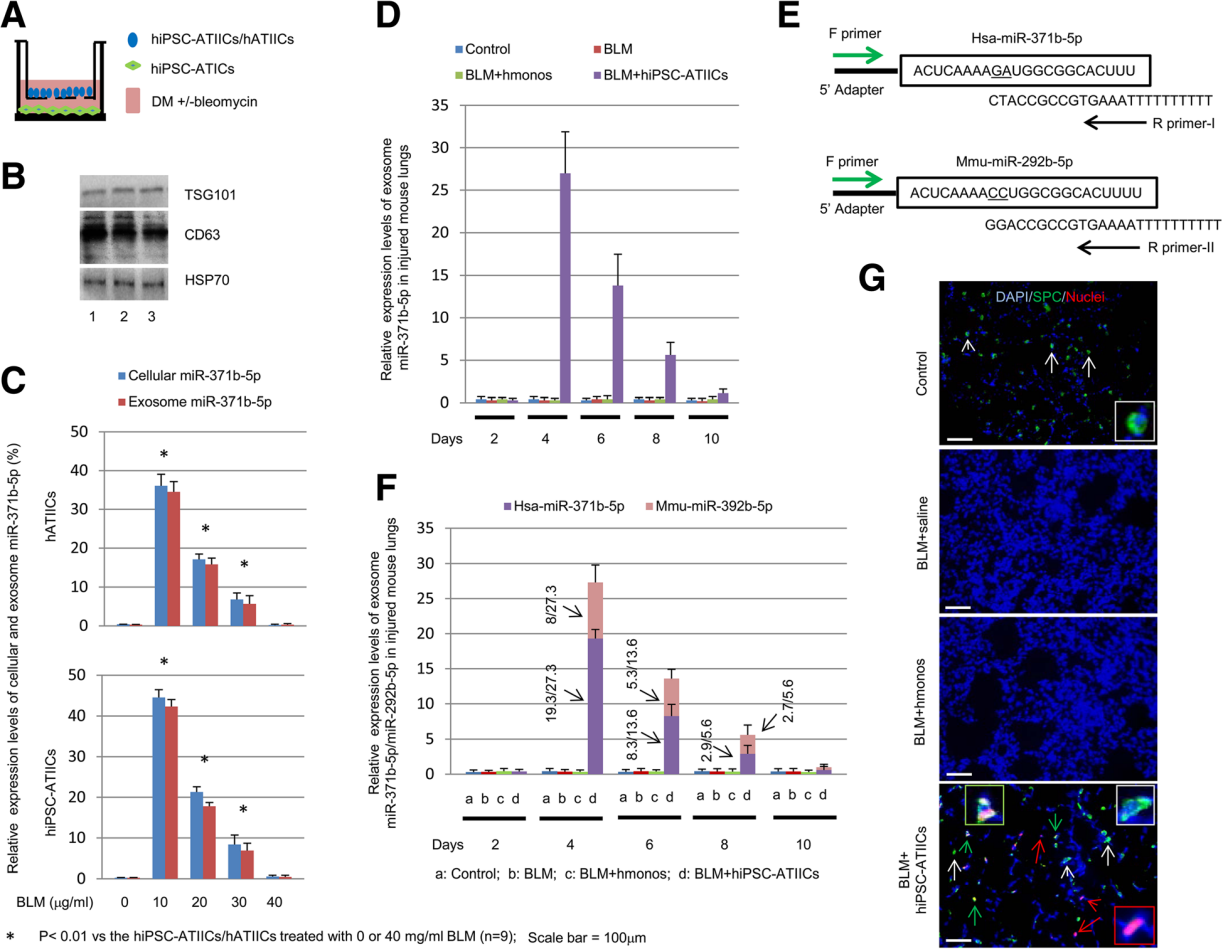
ATIICs express exosome miR-371b-5p in response to injury. **a** Schematic strategy of co-cultures of hiPSC-ATIICs or hATIICs with hiPSC-ATICs in six-well transwell plates using exosome-depleted DM with and without BLM. **b** Western blotting of TSG101, CD63 and HSP70 in exosomes derived from cultured A549 (1), hiPSC-ATIICs (2) and hATIICs (3). QRT-PCR analysis of expression levels of cellular and exosome miR-371b-5p in the cultures of ATIIC phenotypes treated with various doses of BLM (**c**) as well as the level of exosome miR-371b-5p in the lavage samples isolated from BLM-treated mouse lungs transplanted with hiPSC-ATIICs or hmonos (**d**) by using ABM primer. The expression levels were normalized to 18 s and presented as percentage of the cellular or exosome miR-371b-5p level of A549 cells. **e** Schematic structures to show the sequences of miR-371b-5p (*top*), in which two underlined nucleotides are not identical in miR-292b-5p (*bottom*), as well as the sequences of miR-371b-5p-specific reverse primer (R-primer-I) and miR-292b-5p-specific reverse primer (R-primer-II). **f** QRT-PCR analysis of expression levels of exosome miR-371b-5p and miR-292b-5p in BLM-treated lungs with or without transplantation by using R-primer-I or R-primer-II. The expression levels were normalized to 18 s and presented as a ratio to the ABM primer detected ‘miR-371b-5p’ expression level as indicated. **g** Immunofluorescent staining of representative lung sections from BLM-challenged mice with or without transplanted hiPSC-ATIICs or hmonos by using a mouse anti-human nuclei antibody and rabbit anti-human proSPC antibody with DAPI counterstaining. The human nuclei antibody recognizes human cells (*red*) in mouse lungs; the anti-human proSPC stains both mouse and human SPC-expressing ATIICs (*green*). Many nuclei^-^SPC^+^mouse ATIICs (indicated by *white arrows*) were identified in control and BLM-treated lungs transplanted with hiPSC-ATIICs, but not in the BLM-treated lungs receiving hmonos or saline. The nuclei^+^SPC^+^ human ATIICs (indicated by *green arrows*) were found only in the BLM-treated lungs transplanted with hiPSC-ATIICs. Some nuclei^+^ cells were SPC negative (indicated by *red arrows*), suggesting that they were ATICs that had been differentiated from the transplanted hiPSC-ATIICs. A magnified view of a mouse ATIIC (indicated by the *white arrowhead*), a human ATIIC (indicated by the *green arrowhead*), or an ATIC (indicated by *red arrowhead*) is shown in the corresponding image. *BLM* bleomycin, *DAPI* 4′,6-diamidino-2-phenylindole, *DM* differentiation medium, *hATIICs* human primary ATIICs, *hiPSC-ATIICs* human induced pluripotent stem cell-derived ATIICs, *hmonos* human peripheral blood monocytes, *SPC* surfactant protein C

Our previous studies reveal a pivotal role of ATIIC-derived signaling in promoting re-epithelialization of injured alveoli [1, 2]. Here we aimed to test whether normal ATIICs can express an active signaling in response to injury via exosome miR-371b-5p-mediated cell-to-cell communication. Initially, we did not observe miR-371b-5p expression in the cultures of hiPSC-ATIICs or hATIICs treated with BLM (Additional file 1: Figure S4). Considering that ATIC-derived factors may be required to stimulate ATIICs’ function in response to injury, we established an ATIC/ATIIC co-culture model using transwell plates to mimic the injured alveolar environment (Fig. 5a). ATICs and ATIICs (hiPSC-ATIICs or hATIICs) in the co-culture system were exposed to various doses of BLM for 24 hours, and then the cells and exosomes were separately isolated from ATIIC chambers for miRNA preparation. Like A549-derived exosomes, the hiPSC-ATIIC- and hATIIC-derived exosomes expressed exosome-specific markers TSG101, CD63, and HSP70 (Fig. 5b). QRT-PCR was performed to analyze miR-371b-5p expression (as described in “Methods”), and the expression levels of cellular and exosome miR-371b-5p derived from A549 cells were used as control index. As shown in Fig. 5c, neither cellular nor exosome miR-371b-5p were derived from control hiPSC-ATIICs or hATIICs. In contrast, hiPSC-ATIICs and hATIICs were induced to express cellular and exosome miR-371b-5p to significantly detectable levels (Fig. 5c, represented as percentages of those derived from A549 cells) after treated with BLM at 10, 20, or 30 μg/ml. Both cellular and exosome expressions of miR-371b-5p were consistently induced in these two ATIIC phenotypes, with more robust induction at lower BLM concentrations: approximately 43% (hiPSC-ATIICs) or approximately 35% (hATIICs) at 10 μg/ml BLM; 20% (hiPSC-ATIICs) or approximately 17% (hATIICs) at 20 μg/ml BLM, and; approximately 8% (hiPSC-ATIICs) or approximately 6% (hATIICs) at 30 μg/ml BLM. However, miR-371b-5p expression by hiPSC-ATIICs and hATIICs was barely detected after treatment with 40 μg/ml BLM, possibly due to seriously damaged ATIICs so that they failed to respond to injury in the cultures.

We also examined the capacity of ATIICs to derive exosome miR-371b-5p in vivo in injured microenvironment by transplanting hiPSC-ATIICs into a BLM-induced acute lung alveolar injury model, using immune-deficient SCID mice to avoid graft rejection [40, 41]. As previously reported by our laboratory [1, 2], 8–10-week-old female SCID mice were exposed to BLM (3.5 units/kg) 2 days before transplantation with hiPSC-ATIICs (1 × 10^6^ in 50 μl of saline). After exposure to this relatively high dose of BLM, injured alveolar ATIICs barely survive [1, 2]. Thus, transplanted hiPSC-ATIIC-derived signaling as well as its capacity to promote survival of endogenous ATIICs can be clearly evaluated. To demonstrate the specificity of hiPSC-ATIIC-derived signaling, BLM-challenged mice were also transplanted with same number of hmonos. Lavage exosomes, which expressed exosome-specific markers TSG101, CD63, and HSP70 (data not shown), were isolated from BLM-challenged lungs with and without transplantation on days 2, 4, 6, 8, and 10 for miRNA preparation. Initially, miR-371b-5p primers (ABM Inc.) were used to analyze hiPSC-ATIIC-derived exosome signaling. Our results showed that no exosome miR-371b-5p was expressed in control lungs as well as BLM-challenged lungs at each time point (Fig. 5d). In comparison, a significant high level of exosome miR-371b-5p was detected in BLM-challenged lungs 2 days after transplantation with hiPSC-ATIICs (on day 4). As no exosome miR-371b-5p was expressed in BLM-challenged lungs transplanted with hmonos, our data demonstrated an induced expression of hiPSC-ATIIC-specific miR-371b-5p in response to alveolar injury (Fig. 5d). However, as many miRNAs and miRNA recognition (target) motifs are very well conserved in many animal species [42–45], it is rather likely that the ABM primers used for QRT-PCR analysis of the human miR-371b-5p expression can also detect its mouse homolog [46]. We therefore compared the sequence of miR-371b-5p with that of its mouse homolog. As shown in Fig. 5e, miR-371b-5p and its mouse homologous miR-292b-5p do share approximately 91% sequence homology. For clarification, we designed two reverse primers, R-primer-I and R-primer-II (Fig. 5e), to specifically analyze miR-371b-5p and miR-292b-5p expression, respectively. As shown in Fig. 5e, the isolated Exo-miRs were ligated to an RNA 5′ adaptor (Qiagen, Hilden, Germany) before QRT-PCR analysis by using a universal forward primer (Qiagen) and a specific reverse primer (R-primer-I or R-primer-II). The ABM primer detected ‘miR-371b-5p’ expression level was used as control index because the ABM primers are expected to detect both miR-371b-5p and miR-292b-5p (also demonstrated by our preliminary experiments, data not shown). Consistent with the result shown in Fig. 5d, the exosomes isolated from control lungs, BLM-challenged lungs as well as BLM-challenged lungs transplanted with hmonos did not express miR-371b-5p and miR-292b-5p at each time point (Fig. 5f). The fact that no miR-371b-5p/miR-292b-5p expression was detected in control lungs could be because its expression is only restricted to early developmental stage [46]. Interestingly, BLM-challenged lungs expressed significant levels of exosome miR-371b-5p and miR-292b-5p 2 days after transplantation with hiPSC-ATIICs (on day 4, Fig. 5f, represented as a ratio to the ABM primer detected ‘miR-371b-5p’ expression level). These data indicated that the transplanted hiPSC-ATIICs were induced to express miR-371b-5p^+^Exo-miRs in response to alveolar injury in vivo just as in the in vitro experiments. Since mATIICs can also be induced to proliferate by miR-371b-5p^+^Exo-miRs (A549-Exo-miRs, Fig. 2a), the expression of mouse exosome miR-292b-5p in the BLM-challenged lungs transplanted with hiPSC-ATIICs, but not in control lungs, BLM-challenged lungs or BLM-challenged lungs transplanted with hmonos, suggests that the hiPSC-ATIIC-derived miR-371b-5p^+^Exo-miRs may promote survival/recovery of endogenous injured ATIICs, which subsequently release miR-292b-5p^+^Exo-miRs and participate in the repair process. In support of the capacity of mATIICs to release the miR-292b-5p^+^Exo-miRs, the miR-292b-5p expression was demonstrated in BLM-treated mATIIC cultures (Additional file 1: Figure S5). Negative expression of exosome miR-292b-5p in the BLM-treated lungs (without ATIIC transplantation) may reflect the severity of endogenous ATIIC injury. As indicated in Fig. 5d and f, expression of hiPSC-ATIIC-derived exosome miR-371b-5p as well as endogenous exosome miR-292b-5p significantly decreased in BLM-challenged lungs on day 6 and was barely detected on day 10 when injured alveoli were almost completely repaired (data not shown). Such decreased expression of exosome miR-371b-5p/miR-292b-5p over time to the end point (day 10) may reflect the process of recovery of injured alveoli after transplantation. Consistently, the endogenous ATIICs were severely damaged after exposure to this high dose of BLM, and in comparison to control lungs, only a few mouse ATIICs (nuclei^-^SPC^+^) survived in the BLM-challenged lungs receiving saline or hmonos (Table 1 and Fig. 5g). Remarkably, numerous mouse ATIICs were observed in the BLM-challenged lungs where transplanted hiPSC-ATIICs (nuclei^+^SPC^+^) had efficiently engrafted (Table 1 and Fig. 5g), differentiated into ATICs (Additional file 1: Table S2), and released exosome miR-371b-5p (Fig. 5d and f). Taken together, our results demonstrate the capacity of ATIICs to derive miR-371b-5p^+^Exo-miRs in vitro and in vivo in response to injury, which may mediate a communication between ATIICs to promote survival and participation of endogenous injured ATIICs in repair of injured alveoli.

**Table 1:** Relative content of human and mouse ATIICs in bleomycin-mouse lung tissue

	SPC^+a^	SPC^+^Nuclei^+b^	SPC^+^Nuclei^-c^	SPC^+^Nuclei^+^ (%^d^)
Saline-SCID	1083 ± 54.1	0	1083	0
BLM-SCID/saline	13.2 ± 3.2	0	13	0
BLM-SCID/hmonos	14.8 ± 5.2	0	15	0
BLM-SCID/hiPSC-ATIICs	991 ± 39.8	227.6 ± 11.8	763.4 ± 32.8	29.8

*Abbreviations*: *ATIICs* alveolar epithelial type II cells, *SPC* ^*+*^ human and mouse ATIICs, *SPC* ^*+*^ *Nuclei* ^*+*^ human ATIICs, *SPC* ^*+*^ *Nuclei* ^*-*^ mouse ATIICs, *SCID* severe combined immunodeficiency, *BLM* bleomycin, *hmonos* human monocytes, *hiPSCs* human induced pluripotent stem cells,

^a^Number of SPC^+^ cells

^b^Number of SPC^+^Nuclei^+^ cells

^c^Number of SPC^+^Nuclei^-^ cells in the same counted area of 2000 DAPI-stained cells in BLM-mouse lung tissue ^d^Ratio of hiPSC-ATIICs to mouse ATIICs

### Expression of exosome miR-371b-5p in lung lavage of patients with acute pneumonia

**Fig. 6:**
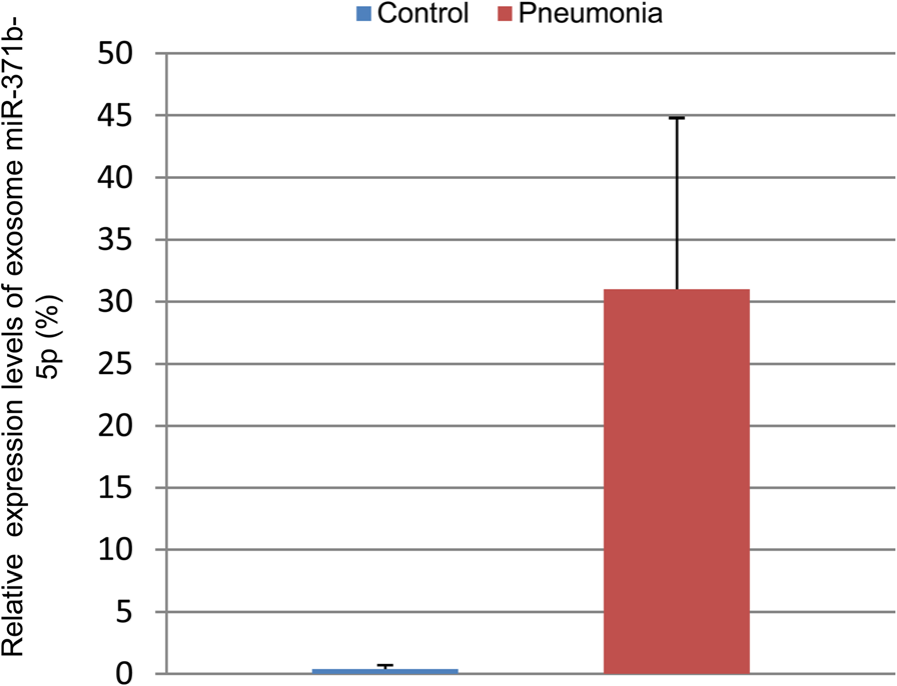
Exosome miR-371b-5p expressed in lungs of patients with acute pneumonia. Total exosome RNA (0.1 μg) isolated from each lung lavage sample of patients with acute pneumonia or without pulmonary disorders (control) was analyzed for miR-371b-5p expression by QRT-PCR with ABM primer and represented as percentages of an exosome miR-371b-5p level of A549 cells after being normalized to 18 s control (*n* = 9)

To examine if exosome miR-371b-5p is expressed in vivo, we also collected lavage samples of patients with acute pneumonia and without any pulmonary disorders for Exo-miR preparation, and analyzed the miR-371b-5p expression by QRT-PCR. Consistent with the in vitro and in vivo data described above, significant expression of exosome miR-371b-5p was detected in lavage samples of patients with acute pneumonia, but not in samples collected from patients without pulmonary disorders (Fig. 6). Taken together, these data strongly support our hypothesis that ATIIC-derived exosome miR-371b-5p may play a critical role in maintaining alveolar homeostasis in response to injury.

## Discussion

Signal pathways derived from niche supporting cells are critical in the regulation of tissue stem/progenitor cell function during development and regeneration. In the lung, several stem/progenitor cell types reside in anatomically diverse regions [47–51] and likely respond to distinct niche signals for maintenance or repair of functionally and structurally different airway and alveolar epithelia. Identifying niche signaling pathways that regulate lung stem/progenitor cell behavior is key to developing new therapies for targeted activation of endogenous regenerative capacity. However, although extensive effort has been devoted to identifying lung stromal cell-derived niche factors that direct airway stem/progenitor cell fate [52–57], alveolar niche signal pathways as well as their molecular bases controlling alveolar progenitor ATIIC behavior remain elusive.

We hypothesize that alveolar progenitor ATIICs may derive a functional niche signaling to robustly promote re-epithelialization of injured alveoli. In this present study, we used a differentiating model of pluripotent stem cells to screen ATIIC phenotype-derived signaling and showed that ATIIC line A549-derived exosomes, but not those derived from human normal ATIIC types (hiPSC-ATIICs and hATIICs), mediated a cell-to-cell communication using miRNAs to promote ATIIC-specific proliferation in the pluripotent stem cell-derived cultures. MiRNA expression profiling analysis shows that A549 cells express a phenotype-specific Exo-miR signature. To identify the Exo-miR signaling, we tested each individual A549-specific Exo-miR in an unbiased manner and demonstrated for the first time that exosome miR-371b-5p is the key factor to promote ATIIC-specific proliferation in the pluripotent stem cell-derived cultures by using PTEN as its direct downstream target to activate the PI3K/Akt signaling pathway. Although A549 cell-derived exosomes express a high level of miR-21, which can also target PTEN [58–60], use of miR-21 inhibitor vector did not affect the capacity of A549 cell-derived Exo-miRs to promote ATIIC-specific proliferation (data not shown), suggesting that exosome miR-21 is not involved in this process. This is because miR-21 is highly expressed by normal ATIIC phenotypes, hiPSC-ATIICs (Fig. 2b) and hATIICs (data not shown), at similar levels. Expression of miR-21 may be required for ATIICs to maintain their basic proliferation capacity as a progenitor cell type. In fact, miR-371b-5p^+^Exo-miRs promote the cell proliferation in the cultures of ATIIC phenotypes, but not in those of non-ATIIC phenotypes, suggesting more Exo-miRs involved in the complex process of induced ATIIC-specific proliferation in the pluripotent stem cell-derived cultures. How miR-371b-5p works together with other Exo-miRs in a coordinating manner to promote ATIIC-specific proliferation is unknown and merits further investigation, which may provide significant insight into the mechanisms underlying alveolar development/regeneration.

We observed that the BLM-treated hiPSC-ATIICs and hATIICs expressed exosome miR-371b-5p, suggesting a possibility that ATIIC-derived exosome miR-371b-5p may serve as an alveolar niche signaling in response to lung injury. To further test this, we transplanted hiPSC-ATIICs into acute injured mouse lungs, in which the repair capacity of endogenous ATIICs had been eliminated by BLM. As previously reported by our laboratory [1, 2], we showed that the transplanted hiPSC-ATIICs can efficiently engraft and differentiate into ATICs for repair of injured alveoli. Interestingly, the BLM-treated lungs transplanted with hiPSC-ATIICs, but not the BLM-treated lungs and BLM-treated lungs transplanted with hmonos, expressed a significant level of human ATIIC-specific exosome miR-371b-5p, demonstrating the ability of the engrafted hiPSC-ATIICs to derive an Exo-miR signaling in response to lung injury in vivo. Consistent with this finding, a significant level of exosome miR-371b-5p was also detected in injured human lungs. As ATIICs are active in secreting and recycling of vesicle-rich surfactant [17, 61–63], exosome-mediated communication between ATIICs is thought to be an efficient and robust means to exchange miRNAs for maintaining alveolar homeostasis; in other words, the hiPSC-ATIIC-derived exosome miR-371b-5p in the injured lungs could be efficiently and specifically transferred to neighboring and distant mouse ATIICs to promote the endogenous repair capacity. While we do not have the direct evidence to show Exo-miR-mediated communication between ATIICs in vivo yet, we observed numerous mouse ATIICs survived in BLM-injured lungs that expressed human ATIIC-specific exosome miR-371b-5p, but only a few in the BLM-exposed lungs that were exosome miR-371b-5p negative. Interestingly, our data suggest that those survived mATIICs may also produce miR-292b-5p, the mouse homolog of miR-371b-5p, to participate in the process of re-epithelialization. Collectively, our data strongly suggest that ATIIC-derived exosome miR-371b-5p may contribute to the alveolar signal microenvironment in response to injury, playing a critical role during the re-epithelialization of injured alveoli, and thus may provide a novel target to develop treatment for currently incurable lung diseases.

## Conclusions

In summary, we have identified the ATIIC phenotype-derived exosome miR-371b-5p as a key factor that promotes ATIIC-specific proliferation by using PTEN as its direct downstream target to orchestrate the PI3K/Akt signaling pathway. The ATIIC phenotype-derived exosome miR-371b-5p may serve as a niche signaling to promote re-epithelialization of injured lung alveoli in response to injury, and thus provide a novel target to develop treatment for repair of injured alveoli.

## Additional file

Additional file 1: Selection and characterization of hiPSC-, hESC-, and mESC-derived ATIICs. (PDF 482 kb)

## Abbreviations

ATICs: Alveolar epithelial type I cells
ATIICs: Alveolar epithelial type II cells
BLM: Bleomycin
BrdU: Bromodeoxyuridine
CM: Conditioned medium
DAPI: 4′,6-Diamidino-2-phenylindole
DM: Differentiation medium
Exo-miRs: Exosome miRNAs
hATIICs: human primary ATIICs
hESCs: Human embryonic stem cells
hiPSC-ATIICs: Human induced pluripotent stem cell-derived ATIICs
hmonos: Human peripheral blood monocytes
mATIICs: mouse primary ATIICs
miRNAs: MicroRNAs
PIP3: Phosphatidylinositol-(3,4,5)-trisphosphate
SPC: Surfactant protein C
SPCP/NEO^R^: SPC promoter/neomycin^R^ transgene
Wt: Wild-type
