# Supplementary material for: Exosome miR-371b-5p promotes proliferation of lung alveolar progenitor type II cells by using PTEN to orchestrate the PI3K/Akt signaling
Source: Stem Cell Res Ther. 2017 Jun 8;8:138. doi: 10.1186/s13287-017-0586-2 (PMC5465462; doi:10.1186/s13287-017-0586-2)
Supplement: Additional file 1: — Selection and characterization of hiPSC-, hESC-, and mESC-derived ATIICs. (PDF 482 kb) [file 13287_2017_586_MOESM1_ESM.pdf]

Supplementary Material

Table S1. Relative content of hiPSC-ATIICs in G418-selected cultures of differentiated hiPSC-26B

	G418 selection	SPC^{+} cells	SPC^{-} cells	SPC^{+} cells %*
H9.2	-	131	869	13.1±0.85
hiPSC-26B	-	137	863	13.7±0.73
hiPSC-26B	+	995	5	99.5±3.12

* The percentage of SPC positive cells was determined by visually counting 1,000 cells based on DAPI staining in the differentiated cultures on day 14.

Abbreviations: ATIICs, alveolar epithelial type II cells; hiPSC, human induced pluripotent stem cell; SPC, surfactant protein C.

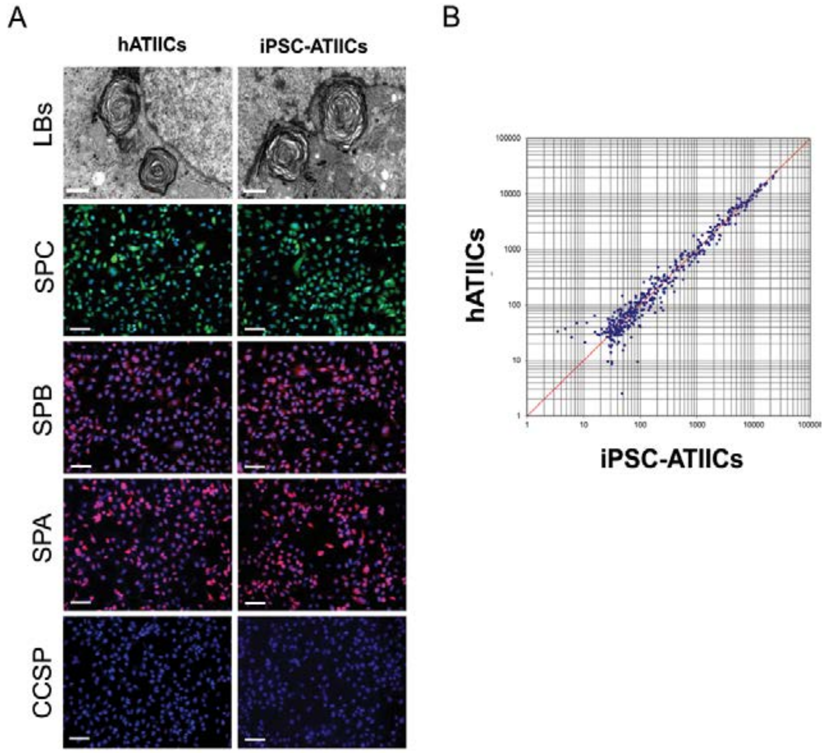

Figure S1. Characterization of hiPSC-ATIICs. A human iPSC line, hiPSC-26B [1], was cultured on Matrigel-coated plates in DM (differentiation medium) containing G418 (20 μg/ml, Gibco Invitrogen) for 14 days, and the selected hiPSC-ATIICs (hiPSC-derived ATIICs) were characterized. (A) The hiPSC-ATIICs and human primary ATIICs (hATIICs) express lamellar bodies (LBs, scale bar = 0.5 μm) and surfactant proteins A, B, and C, but not CCSP (scale bar = 200 μm). (B) Scatter plot of miRNA array analysis shows that hiPSC-ATIICs exhibit hATIIC-specific miRNA expression pattern.

Abbreviations: SPB, surfactant protein B; SPA, surfactant protein A.

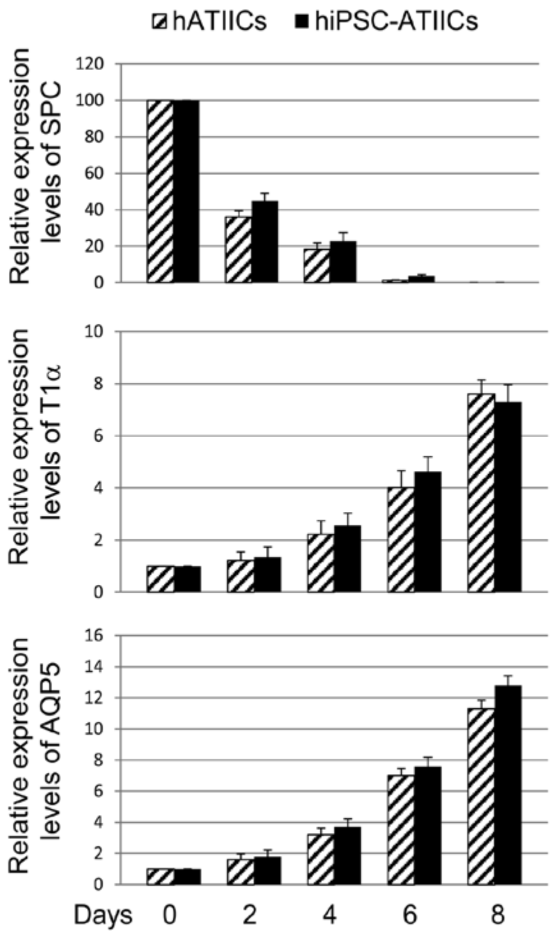

Figure S2. Differentiation of hiPSC-ATIICs into ATICs. $1x10^6$ hiPSC-ATIICs or hATIICs were placed in each well of 6-well plates with DMEM containing 10 % FBS and were left to spontaneously differentiate for up to 8 days. As previously reported [1, 2], QRT-PCR was performed to analyze the expression levels of ATIIC-specific SPC and ATIC-specific markers, T1α and AQP5, in the differentiating cultures of hiPSC-ATIICs on days 0, 2, 4, 6, and 8, using 18S rRNA as endogenous control (n = 8). The expression levels of T1α and AQP5 significantly increased, along with significantly decreased SPC expression over time in the differentiating cultures of hiPSC-ATIICs and hATIICs, indicating the ability of hiPSC-ATIICs to spontaneously differentiate into ATICs *in vitro*.

Abbreviations: ATICs, alveolar epithelial type I cells.

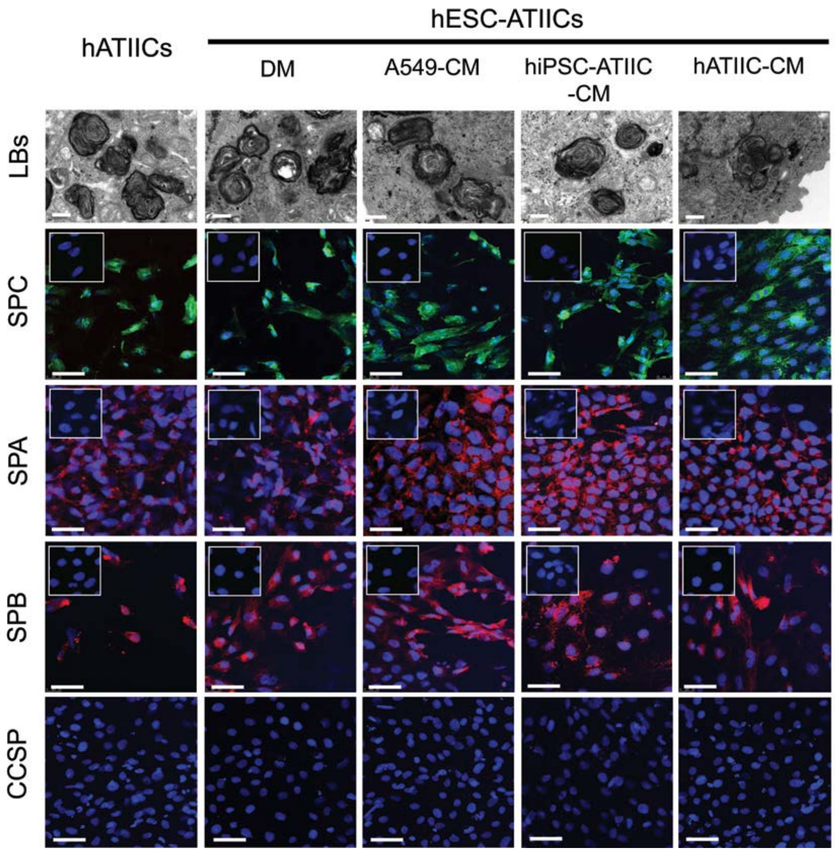

Figure S3. Characterization of hESC-ATIICs. The hESCs, SPCP/NEO.74 [3], were subjected to spontaneous differentiation in DM for 6 days. The derived cultures were then treated with A549-CM, hiPSC-ATIIC-CM or hATIIC-CM for 6 days, before hESC-derived ATIICs (hESC-ATIICs) were selected with G418 (60 μg/ml, overnight) for further characterization. Electron micrographs showed well-developed lamellar bodies (LBs) in hESC-ATIICs selected from both treated and untreated cultures as observed in hATIICs (top panel, scale bar = 0.5 μm). Immunofluorescent staining demonstrated that all G418-selected hESC-ATIICs in both treated and untreated cultures express SPA, SPB and SPC, but not CCSP, like control hATIICs (Scale bar = 50 μm). The isotype staining controls for SPC, SPA or SPB are shown in the corresponding inserts.

Abbreviations: hESCs, human embryonic stem cells.

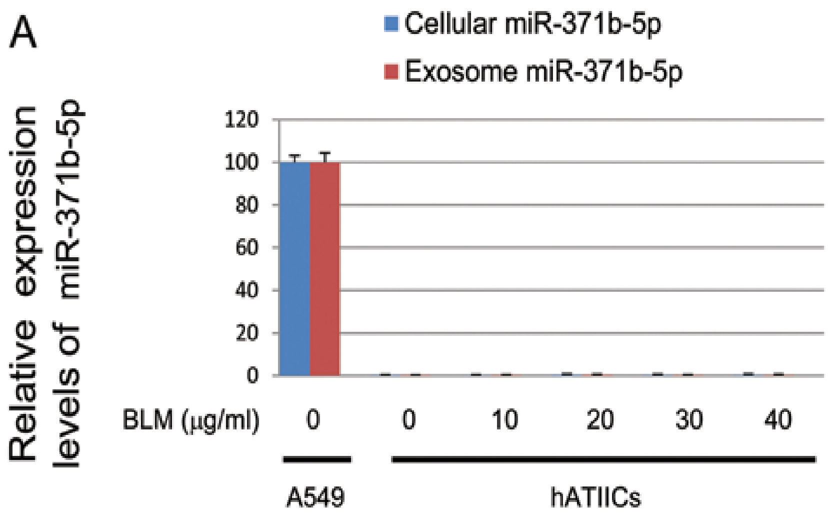

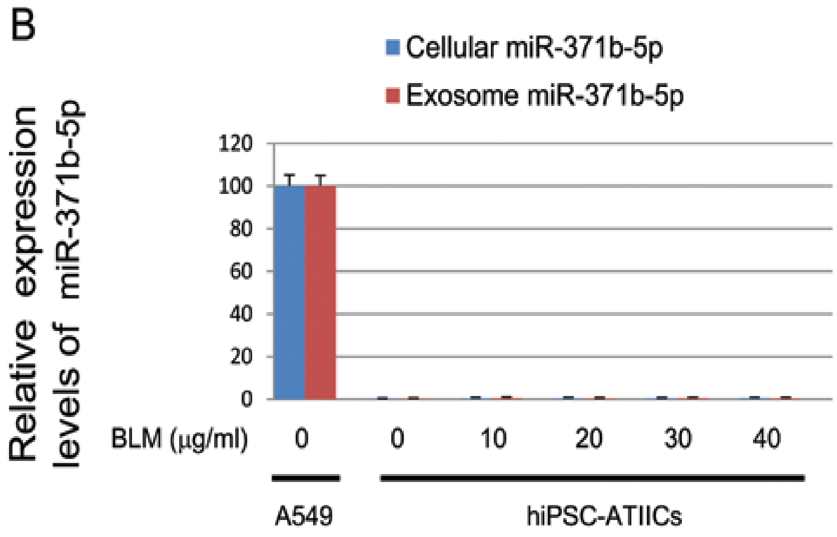

Figure S4. Expression levels of cellular and exosome miR-371b-5p in the BLM-treated cultures of ATIIC phenotypes. The hATIICs (A) and hiPSC-ATIICs (B) were placed at $1X10^6$/well in 6-well plates in DM, and then treated with various doses of BLM (bleomycin, as indicated) for 24 hrs., after which cells and exosomes were separately isolated for miRNA preparation. The expression levels of miR-371b-5p were normalized to 18s and presented as percentage of the cellular or exosome miR-371b-5p level of A549 cells.

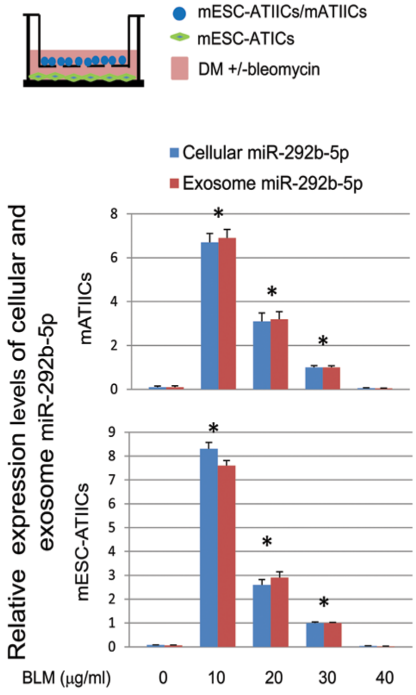

Figure S5. BLM-treated mouse ATIICs express cellular and exosome miR-292b-5p. Schematic strategy of co-cultures of mESC-ATIICs or mATIICs with mESC-ATICs in 6-well transwell plates using exosome-depleted DM with or without BLM (top panel). MESC-ATIICs and mATIICs were prepared as we previously reported [4]. For the co-culture model, $5x10^{5}$ mESC-ATIICs were placed in the lower chamber of each 6-well transwell plate with DMEM containing 10 % FBS for 8 days to generate mESC-ATICs. Then the derived ATICs were switched to exosome-depleted DM, and $1x10^{6}$ freshly isolated mESC-ATIICs or mATIICs were placed in each transwell insert with 1 ml exosome-depleted DM. On the next day, BLM of different dose (10, 20, 30 and 40 µg/ml) was added to the medium to induce injury for 24 hours before exosome and Exo-miR isolation. QRT-PCR was performed to analyze expression levels of cellular and exosome miR-292b-5p in these BLM-treated cultures of mATIICs and mESC-ATIICs. The expression levels were normalized to 18s.

Abbreviations: mESC-ATIICs, mouse embryonic stem cell-derived ATIICs; mESC-ATICs, mESC-derived ATICs.

Table S2. Relative content of human derived ATICs in BLM-treated mouse lung tissues

	$Nuclei^+$	$T1a^+/Nuclei^+$ (#)	$T1a^+/Nuclei^+$ (%)
Saline-SCID	-	-	-
BLM-SCID	-	-	-
BLM-SCID/hmonos	-	-	-
BLM-SCID/hiPSC-ATIICs	500	181 ± 11.4	36.2

$Nuclei^+$: human derived cells; $T1a^+$: ATICs (alveolar epithelial type I cells); $T1a^+/Nuclei^+$, human ATICs.

#: number of $T1a^+/Nuclei^+$cells in the counted area of 500 $Nuclei^+$cells; %: percentage of human ATICs that had derived from human ATIICs.

Abbreviations: BLM, bleomycin; SCID, severe combined immunodeficiency; hmonos, human monocytes; ATIICs, alveolar epithelial type II cells; hiPSCs, human induced pluripotent stem cells.

References

1. Yan Q, Quan Y, Sun H et al. *A site-specific genetic modification for induction of pluripotency and subsequent isolation of derived lung alveolar epithelial type II cells.* Stem Cells 2014; **32**:402-13.
2. Wang D, Morales JE, Calame DG, Alcorn JL, Wetsel RA. Transplantation of human embryonic stem cell-derived alveolar epithelial type II cells abrogates acute lung injury in mice. Mol Ther, 2010; **18**: 625-34.
3. Wang D, Haviland DL, Burns AR et al. *A pure population of lung alveolar epithelial type II cells derived from human embryonic stem cells.* Proc Natl Acad Sci U S A 2007; **104**:4449-54.
4. Sun H, Quan Y, Yan Q, Peng X, Mao Z, Wetsel RA, Wang D. *Isolation and Characterization of Alveolar Epithelial Type II Cells Derived from Mouse Embryonic Stem Cells.* Tissue Eng Part C Methods, 2014; **20**: 464-72.
